# Narcissistic traits as mediators in the relationship between parenting styles and nonconsensual nonmonogamy in university students

**DOI:** 10.3389/fpsyg.2026.1885928

**Published:** 2026-07-29

**Authors:** Suany Pineda-Sandoval, Cesia Bustillo-Núñez, Hanny Gómez, Jennifer Chevez, Yeyson Sosa, Marta Kowal, Miguel Landa-Blanco

**Affiliations:** 1 School of Psychological Sciences, National Autonomous University of Honduras, Tegucigalpa, Honduras; 2 IDN Being Human Lab—Institute of Psychology, University of Wrocław, Wrocław, Poland

**Keywords:** infidelity, narcissism, nonconsensual nonmonogamy, parenting styles, unfaithful behavior, unfaithfulness

## Abstract

**Introduction:**

Despite its prevalence and well-documented negative consequences, the factors contributing to infidelity remain poorly understood. This study aimed to shed more light on these matters by examining the interplay between infidelity, perceived childhood parenting styles, and subclinical narcissistic traits in males and females.

**Methods:**

Participants were 399 students from the National Autonomous University of Honduras.

**Results:**

Forty-five percent of male participants reported having been sentimentally involved with someone other than their partner, compared with 26% of female participants. Additionally, 15% of men reported having had sex with someone other than their partner, compared with 5% of women. Among females, none of the included parenting or personality variables significantly predicted infidelity. Among men, recalled parental emotional warmth was directly associated with lower infidelity-related behaviors and desires, but indirectly associated with higher infidelity-related behaviors and desires through higher narcissistic traits. Furthermore, parental rejection in males was positively associated with infidelity-related behaviors. The distinct path models observed for males and females suggest sex-specific mechanisms through which parenting styles may be related to adult infidelity behaviors.

**Discussion:**

We conclude that longitudinal research is needed to clarify these mechanisms and guide infidelity prevention efforts.

## 1 Introduction

In many cultures, romantic relationships are often considered a bedrock of human functioning (Buss, 2019; Fletcher, 2015). They are a source of great satisfaction and pleasure, but can also cause significant dissatisfaction and stress (Gigy and Kelly, 1993). One of the factors that significantly destabilizes romantic relationships is infidelity, which has raised substantial scientific interest. This introduction outlines the conceptual background and prevalence of infidelity, followed by evidence on its main psychological and developmental correlates, including sex differences, parenting styles, and subclinical narcissistic traits. It concludes by addressing the cultural context of Honduras and presenting the aims and hypotheses guiding the present research.

### 1.1 What is infidelity?

Infidelity is defined as the violation of a relationship agreement, whether emotional, sexual, or both, toward a spouse or other sexual partner (Balon, 2016). Some scholars highlight that infidelity involves a partner's breach of relational norms concerning the nature of interpersonal and sexual interactions with others (Drigotas et al., 1999), thus encompassing any behavior that contravenes the expectations established within a relationship and its exclusivity (Moller and Vossler, 2015; Morrissey et al., 2018). Importantly, such behaviors occur without the partner's knowledge or consent, distinguishing them from consensual non-monogamous arrangements (Scoats and Campbell, 2022). These infidel behaviors can include sexual intercourse and all extradyadic sexual activities, as well as emotional and deep connections with a significant other outside of the romantic relationship (Moller and Vossler, 2015).

Extradyadic sexual activities may comprise a range of sexual or erotic behaviors, such as masturbation, sexual play, kissing, cybersex, the use of online pornography, and engaging in sexual fantasies with someone other than one's partner (Moller and Vossler, 2015). Complementarily, emotional betrayal manifests through a deep emotional bond with someone other than one's partner, or through actions that involve investing time, attention, and the exchange of secrets or complaints about the primary partner, without the need for sexual contact (Moller and Vossler, 2015; Morrissey et al., 2018).

Similarly, infidelity among young adults is often driven by unmet interdependence needs within their primary relationships, which may lead them to seek emotional or relational fulfillment through alternative partners (Norona et al., 2018). However, a significant proportion of young adults also reported motives that were not directly related to interdependence, including opportunities for intimacy with an affair partner while under the influence of alcohol, physical or emotional attraction toward the affair partner, and the excitement, novelty, or thrill associated with the infidelity experience (Norona et al., 2018).

### 1.2 Prevalence of infidelity

Infidelity is a widespread phenomenon, with estimates suggesting it occurs in approximately one out of every four marriages (Coop Gordon and Mitchell, 2020). However, prevalence rates vary across populations (Murphy et al., 2024). A representative sample of 2,579 American adults indicated that 25.7% (*n* = 663) had experienced a partner's infidelity (Hoy and Oh, 2024). Among 790 married Mexican adults, 17% (*n* = 134) reported having engaged in infidelity themselves (Moral-de-la-Rubia, 2020). Among university students, rates of infidelity are notable. In a sample of 500 American students, 10.6% (*n* = 53) reported having been unfaithful during an exclusive relationship, with only 25.93% disclosing the infidelity to their partner (Graham et al., 2016). Similarly, in a sample of 216 Mexican university students, 25% (*n* = 54) reported a desire to engage in emotional infidelity without sexual involvement, whereas 4.6% (*n* = 10) admitted to sexual contact with someone outside the relationship; some participants also reported both emotional and sexual desires toward others (Peña Peña and Mendoza Gonzalez, 2022). Additionally, the rise of technology and dating apps has introduced new opportunities for infidelity. In a study conducted in the United Kingdom, 15.31% (*n* = 51) of 333 participants reported using dating apps while in a committed relationship (Nascimento et al., 2024), highlighting the role of digital platforms in facilitating extradyadic behaviors (Alexopoulos et al., 2020).

### 1.3 Factors related to infidelity

Building on evidence that multiple psychological and developmental factors influence infidelity, the present study also considered the role of sex differences, parenting styles, and subclinical narcissistic traits in shaping infidelity-related behaviors. Regarding the first topic, men tend to engage in infidelity more frequently than women (Vail-Smith et al., 2010). Men were more likely to report engaging in sex for physical desirability, pleasure, experience seeking, and self-esteem enhancement. They also showed higher endorsement of motives related to novelty, status, and the “thrill of the forbidden.” Women, by contrast, tended to report higher motivation for love, commitment, and emotional connection. These differences suggest that utilitarian and sensation-seeking factors influence men more, whereas women place greater emphasis on the relational and affective dimensions of sexual behavior (Kelberga and Martinsone, 2021). From an evolutionary perspective, differences in infidelity between men and women can be explained by the sexual strategies theory (Buss and Schmitt, 1993), which proposes that reproductive pressures have shaped distinct mating behaviors in each sex. Because men can impregnate multiple women simultaneously, they have evolved strategies oriented toward sexual variety and short-term mating to maximize reproductive success. In contrast, women's limited biological reproductive capacity has favored selectivity and long-term partnerships that ensure stability and protection for offspring. These evolutionary dynamics help explain men's greater tendency toward sexual infidelity and women's deeper emotional investment in relationships.

As a second related factor, prior research also points out that parenting style may be another factor that is related to infidelity. Parenting style has been defined as a set of parental attitudes and behaviors that create an emotional climate that influences a child's development, socialization, and wellbeing, with lasting effects that can extend into romantic relationship patterns in adulthood (Janovsky et al., 2020). It includes both goal-directed parenting practices (such as roles and discipline) and spontaneous, non-goal-oriented behaviors (such as gestures, changes in tone of voice, or emotional expressions). Furthermore, parenting styles are considered a characteristic of the child's social environment, independent of the child's traits, and can influence the effectiveness of their openness to socialization (Darling and Steinberg, 1993).

Parenting styles may be classified into three types: warmth, rejection, and overprotection (Arrindell et al., 1999). Emotional warmth is characterized by praise, physical affection, or other forms of emotional support. In contrast, rejection is characterized by hostility, punishment, and derogation, and is associated with an increase in externalizing problems in children (Barbot et al., 2014; Fuemmeler et al., 2012; Ju et al., 2021; Sobrinho et al., 2016; Stright and Yeo, 2014). On the other hand, parental overprotection, defined by intrusive control, excessive supervision, rigid expectations, and strict limit-setting (Arrindell et al., 1999), has been linked to insecure attachment in adulthood (Zain et al., 2023), which may increase the likelihood of infidelity (Ghiasi et al., 2024). These early patterns of parenting behavior play a crucial role in shaping attachment styles that persist into adulthood. For instance, parenting styles that lack warmth, apply excessive control, or show inconsistency tend to promote anxious or avoidant attachment. When these attachment patterns persist into adulthood, they often weaken relationship security and heighten the risk of maladaptive relational behaviors such as infidelity (Ghiasi et al., 2024).

Additionally, recent studies suggest that young people's perceptions of the parenting they received directly influence the formation of an inflated and unstable self-concept (Farzand et al., 2021). In turn, this self-concept is an essential factor in the development of subclinical narcissistic traits: youth who perceive parenting characterized by overvaluation or lack of boundaries may construct an exaggerated yet vulnerable self-image, which fosters the emergence of specific narcissistic characteristics. On the other hand, a study evaluating 328 Australian youths found that overprotection, overvaluation, and indulgent discipline from both maternal and paternal figures toward young individuals may be associated with narcissistic traits (Van Schie et al., 2020). Moreover, maternal discipline was explicitly identified as being related to pathological narcissism. These findings indicate that certain parenting styles, such as overprotection, can significantly influence the development of narcissistic characteristics.

The third factor that may play a role in infidelity behaviors is narcissistic traits. Narcissism is characterized by a sense of entitlement, that is, the belief that one deserves special benefits and attention. People high on narcissism frequently engage in social comparisons, tend to experience more envy, have a greater tendency to seek status, report higher self-esteem, exhibit excessive confidence, and demonstrate a willingness to exploit others for personal gain (O'Reilly and Chatman, 2020; Weinberg and Ronningstam, 2022). A systematic review examined the association between parenting styles and narcissism in youth, concluding that there is a significant relationship between the two variables. In particular, permissive parenting and childhood overgratification were found to be positively correlated with narcissistic traits (Kiliçkaya et al., 2023; Irfan Thalib et al., 2024).

Several studies have shown that these are interrelated. For instance, Altinok and Kiliç (2020) found that more narcissistic individuals express greater intention to be unfaithful and that this relationship is influenced by relationship satisfaction and attachment styles. Specifically, their study with 407 university students from Turkey showed that those with high levels of narcissism tend to report lower satisfaction in their relationships, which increases the infidelity intentions. This finding provides important insight, indicating that narcissism is not only directly associated with infidelity but also affects other emotional aspects that contribute to infidelity.

### 1.4 Consequences of infidelity

Emotional and sexual infidelity can not only significantly harm the relationship, potentially leading to separation or divorce, but also negatively impact the emotional wellbeing of the individuals involved. Such consequences may include depressive symptoms, suicide ideation, lowered self-esteem, and elevated levels of stress and anxiety (Moller and Vossler, 2015; Hoy and Oh, 2024; Azhar et al., 2018; Bozoyan and Schmiedeberg, 2023; Snyder et al., 2012).

However, its adverse mental health effects are not limited to the couple but also extend to their offspring. Children exposed to parental infidelity frequently describe being prematurely placed in caregiving roles, experiencing fear of abandonment and relational instability, enduring long-term psychological distress, and navigating a challenging process of healing and reconstruction (Salih and Chaudry, 2023). Additionally, marital infidelity is associated with increased conflict and aggression within the couple (Coop Gordon and Mitchell, 2020). It also has economic repercussions, as households often report financial losses (Crouch and Dickes, 2016). It is also worth noting that, from an evolutionary psychology perspective, it has been reported that men tend to experience greater distress in response to sexual infidelity. In contrast, women are more affected by emotional infidelity, given that each type of betrayal represents a different threat in terms of reproduction and resources (Buss, 2018). For this reason, examining developmental and personality-related correlates of infidelity-related behaviors may help clarify why some individuals are more likely to report these experiences than others.

### 1.5 Honduras cultural context

To better understand the dynamic between parenting styles, subclinical narcissism, and infidelity behaviors, cultural and contextual factors must be considered. In Honduras, infidelity triggers a series of consequences that threaten the health and integrity of couples and individuals. These consequences include violence, suicide, depression, loss of self-esteem, fear, anxiety, stress, and physical and psychological abuse, among others (Ordóñez López, 2022). In this regard, infidelity could be considered a cause of separation in affective and family relationships, having serious consequences on the psychological health of those involved (Azhar et al., 2018; Congreso Nacional, 1984).

Furthermore, the Honduran culture remains marked by a paternalistic and authoritarian parenting model, influenced by a rigid social context that reinforces obedience and submission (Moreno León et al., 2017). The use of corporal punishment, adverse childhood experiences, and endorsement of traditional gender norms are common within the Honduran setting (Landa-Blanco et al., 2026). These cultural factors may further contribute to a higher prevalence of insecure attachment.

### 1.6 Present study

**Figure 1:**
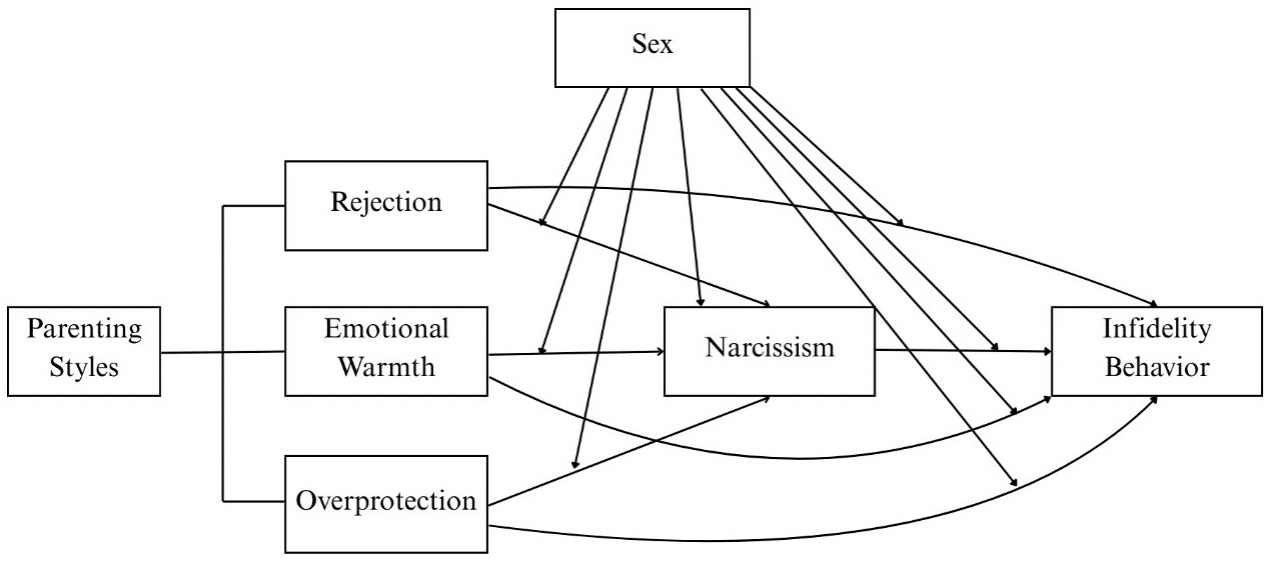
Hypothetical mediation model.

Despite being a topic of psychosocial relevance, the existing literature on the links between parenting style, subclinical narcissism, and infidelity among both males and females remains scarce. Here, we take an integrative perspective and consider how these traits are interrelated; see Figure 1 for the overview of the hypothetical model.

First, research on infidelity indicates that it is associated with multiple individual and relationship factors rather than emerging randomly. For instance, reviews and large empirical studies have identified predictors such as prior infidelity, number of sexual partners, relationship dissatisfaction, low commitment, and attachment-related insecurity. Within this broader literature, parenting styles can be framed as distal developmental influences that may shape later interpersonal expectations, relationship functioning, and behavioral tendencies (Ghiasi et al., 2024; Fincham and May, 2017; Mark et al., 2011). Therefore, our Hypothesis 1 is: Recalled parenting styles experienced in childhood are directly associated with infidelity in adulthood.

Second, parenting experiences are relevant to the development of self-evaluative and interpersonal styles. Parenting dimensions such as warmth, monitoring, and psychological control were related to narcissism, supporting the idea that subclinical narcissistic traits are linked to developmental family experiences (Horton et al., 2006). In adulthood, narcissistic traits, especially when expressed in the relational or sexual domain, have been associated with exploitative interpersonal tendencies and a greater likelihood of infidelity (McNulty and Widman, 2014). Accordingly, subclinical narcissism can be treated as a plausible mediating mechanism linking recalled parenting experiences to infidelity behavior in the present model. Thus, our Hypothesis 2 is: Recalled parenting styles show indirect associations with infidelity behavior through subclinical narcissistic traits.

Third, prior research suggests that infidelity differs by sex not only in prevalence, but also in its correlates and motivations. Studies have shown that men and women may differ in the reasons associated with infidelity and in the pattern of variables linked to it (Barta and Kiene, 2005; Brand et al., 2007), while broader work indicates that infidelity is shaped by multiple demographic, interpersonal, and personality-related factors (Fincham and May, 2017; Mark et al., 2011). Accordingly, it was expected that the associations among recalled parenting styles, narcissistic traits, and infidelity behavior might differ between men and women. Therefore, our Hypothesis 3 is: The associations among recalled parenting styles, narcissistic traits, and infidelity behavior differ between men and women.

The proposed model was specified as an associational mediation framework. Recalled parenting styles were treated as distal developmental experiences, whereas narcissistic traits were examined as personality-related characteristics that may help account for individual differences in infidelity-related behaviors and desires. Because the data are cross-sectional, the model tests theoretically informed indirect associations rather than causal or temporal pathways.

## 2 Materials and methods

### 2.1 Participants

Data was collected in person, in the common areas of the National Autonomous University of Honduras (UNAH) between June 18 and June 30, 2025. Participants were students over 18 years of age and enrolled in one of the university's faculties during the first academic term of 2025. A total of 399 surveys were collected. The average age of participants was 21.9 years (*SD* = 4.61), with ages ranging from 18 to 53 years. The demographic characteristics of the sample are summarized in Table 1.

**Table 1:** Demographic characteristics of the participants.

Variable	Category	Frequency	Percentage
Sex	Female	217	54%
	Male	182	46%
Romantic status	Single	260	65%
	In a relationship	130	33%
	Married	8	2%
	Divorced	1	0%
Sexual orientation	Heterosexual	344	86%
	Bisexual	31	8%
	Homosexual	16	4%
	Other	8	2%

### 2.2 Measures

#### 2.2.1 Demographic questionnaire

Basic demographic information was collected using single-item questions, including age, sex (male, female), romantic status (single, in a relationship, married, divorced, and widowed), and sexual orientation (heterosexual, homosexual, bisexual, and other).

#### 2.2.2 Multidimensional Infidelity Inventory (IMIN)

The inventory, developed by Romero Palencia et al. (2007), originally consisted of 155 items distributed across four subscales addressing infidelity behavior, motivation, conceptualization, and consequences. However, this study employed only the short version of the Infidelity Behavior subscale, which includes 20 items and uses a five-point Likert-type response format: 1 = *Never*, 2 = *Rarely*, 3 = *Sometimes*, 4 = *Frequently*, and 5 = *Always*. Higher scores indicate a higher frequency of engaging in different infidelity behaviors. Sample statements include “I have fallen in love with someone other than my partner” and “I have desired to kiss someone other than my partner.” The IMIN demonstrated excellent internal consistency (ω = 0.95).

#### 2.2.3 Short Egna Minnen av Barndoms Uppfostran (s-EMBU)

This instrument is an abbreviated version of Arrindell et al. (1999), consisting of 23 items with a four-point Likert-type response format: 1 = *No, never*; 2 = Yes, but rarely; 3 = Yes, often; 4 = *Yes, most of the time*. It evaluates perceived parenting styles during one's childhood through three subscales: Rejection, Emotional Warmth, and Overprotection. Higher scores indicate greater intensity of the specific parenting style experienced while growing up. Items include statements such as “My parents wanted to decide how I should dress or look” and “My parents showed me with words and gestures that they loved me.” In the current sample, the s-EMBU exhibited satisfactory reliability values for its dimensions: Rejection (ω = 0.80), Emotional Warmth (ω = 0.87), and Overprotection (ω = 0.79), supporting the subscales' internal consistency.

#### 2.2.4 Short Dark Tetrad (SD4)—Narcissism subscale

This study used the grandiose narcissism subscale of the SD-4 developed by Paulhus et al. (2021) which consists of 7 items and includes statements such as “I have a unique talent for persuading people” and “I am likely to become a future star in some area” (Paulhus et al., 2021). Each item is rated on a 5-point Likert-type response format: 1 = *Strongly Disagree*, 2 = *Disagree*, 3 = *Neutral*, 4 = *Agree*, and 5 = *Strongly Agree*. Higher scores indicate a higher intensity of narcissistic traits. Based on the current data, this subscale demonstrated satisfactory reliability (ω = 0.78), supporting its internal consistency.

### 2.3 Data analysis

Data analysis was conducted using the statistical software JAMOVI (version 2.6.26). Although participants were free to withdraw from the survey at any point, all questions in Google Forms were mandatory, so only fully completed responses could be submitted, ensuring a dataset with no missing data.

In the initial stage, descriptive analyses were performed to characterize the sample. Additionally, the internal consistency of the scales was assessed using McDonald's omega coefficient (ω). Because each participant completed all three s-EMBU subscales, descriptive differences among emotional warmth, overprotection, and rejection were examined using a repeated-measures ANOVA, with parenting-style dimension specified as the within-subjects factor. Estimated marginal means and 95% confidence intervals were reported to describe the pattern of scores across subscales, without interpreting uncorrected pairwise contrasts.

In the second phase, multivariate conditional mediation analyses were conducted using the GLM Mediation Model module to assess direct and indirect relationships. Three models were estimated: an overall model including all participants, and separate models for men and women. In each model, received parenting styles served as the predictor variable, narcissistic traits as the mediator, infidelity behavior as the outcome, and sex assigned at birth as the grouping variable. Furthermore, the proportion of variance explained by the model was quantified using the coefficient of determination (*R*^2^). Finally, all hypothesis tests were evaluated at a 95% confidence level.

A *post-hoc* power analysis was conducted to assess whether the sample size provided sufficient power to detect effects in the mediation model. Power estimates were based on the observed *z*-statistics, standard errors, and sample size. Following Cohen (1992) and Faul et al. (2009), power was calculated using the standard normal approximation formula. In the mediation model, the overall explained variance was converted to an effect size (*f*^2^), and power was estimated using the noncentral *F* distribution.

Mediation analyses were used to examine whether narcissistic traits statistically accounted for indirect associations between recalled parenting styles and infidelity-related behaviors and desires. Because the data were cross-sectional, these models were interpreted as tests of theoretically informed relational patterns rather than as evidence of temporal or causal mechanisms.

### 2.4 Ethical considerations

Data collection was conducted anonymously, without requesting personal information that could identify participants. Before administering the survey, informed consent was obtained, including relevant information about the Research Topic, its objectives, procedures, potential risks, and resources available to minimize them. This document was accessible via a link and a QR code provided in a physical format. Finally, this research, registered under code 202502001, was approved by the Committee on Initial Integral Practice of the School of Psychological Sciences (UNAH) on June 18, 2025.

## 3 Results

### 3.1 Descriptive statistics

Regarding the Infidelity Behavior subscale (*M* = 1.35, *SD* = 0.522, Min = 1, and Max = 4.35), item-level responses were examined in more detail. Eleven percent of the sample endorsed the item “I have had sexual relations with someone other than my partner” (item 9), with men doing so three times as often as women (18% vs. 6%). A comparable pattern emerged for “I have betrayed my partner with another person” (item 17), endorsed by 12% of the sample overall, 18% of men, and 7% of women. Becoming sentimentally involved with someone else while in the current relationship, captured by item 4 (“I have become sentimentally involved with another person”), was considerably more common, reported by 35% of participants overall and by nearly half of the men in the sample (45%) compared with 26% of the women. Desire showed a similar sex gap to behavior: 18% of participants endorsed “I have desired to have sexual relations with someone other than my partner” (item 12), rising to 28% among men against 9% among women. Among the selected items described here, the most frequently endorsed was “I have felt attracted to someone other than my partner” (item 16), reported by 37% of the sample, 43% of men and 32% of women (see Table 2).

**Table 2:** Descriptive analysis for the IMIN—Infidelity behavior subscale.

Item	Male		Female		*M*	*SD*
	*n*	%	*n*	%		
1. Flirting with others.	67	37	60	28	1.48	0.83
2. Multiple romantic partners.	54	30	43	20	1.40	0.81
3. Emotional involvement with others.	62	34	44	20	1.43	0.85
4. Extradyadic sentimental relationship.	82	45	57	26	1.56	0.88
5. Love for others.	51	28	37	17	1.41	0.91
6. Falling in love with others.	43	24	36	17	1.29	0.69
7. Thoughts about others.	92	51	73	34	1.64	0.90
8. Interest in others.	68	37	63	29	1.49	0.82
9. Sexual intercourse with others.	32	18	12	6	1.19	0.62
10. Extradyadic sexual contact.	35	19	15	7	1.21	0.61
11. Desire to kiss others.	58	32	49	23	1.41	0.79
12. Desire for sexual intercourse.	51	28	19	9	1.30	0.75
13. Desire for sexual contact.	52	29	22	10	1.29	0.68
14. Desire for extramarital relations.	33	18	13	6	1.21	0.67
15. Sexual fantasies with others.	47	26	18	8	1.27	0.71
16. Attraction toward others.	79	43	70	32	1.54	0.81
17. Betrayal of a partner.	32	18	16	7	1.18	0.56
18. Deception of a partner.	31	17	16	7	1.17	0.52
19. Extradyadic sexual relations.	27	15	11	5	1.17	0.62
20. Sexual desire for others	47	26	25	12	1.28	0.70

Item-level endorsement was estimated by summing the total number of participants who responded in categories: 2 = rarely, 3 = sometimes, 4 = frequently, and 5 = always. These categories indicate, to some degree, the presence of the infidelity-related behavior assessed in each item. The data presented independently summarizes the number of participants from the male and female samples who endorse each item. For instance, regarding item 1, 37% of men and 28% of women reported flirting with someone other than their partners.

On the other hand, descriptive scores for the s-EMBU subscales were similar for emotional warmth (*M* = 2.49, *SD* = 0.76, and 95% *CI* [2.41, 2.56]) and overprotection (*M* = 2.35, *SD* = 0.56, and 95% *CI* [2.30, 2.41]), while rejection showed a lower mean score (*M* = 1.71, *SD* = 0.58, and 95% *CI* [1.65, 1.77]), *F*_(2,796)_ =158.97, *p* < 0.001. No statistically significant sex differences were found across the parenting style variables: rejection (*p* = 0.098, *d* = 0.17), emotional warmth (*p* = 0.546, *d* = 0.06), and overprotection (*p* = 0.148, *d* = 0.14). Similarly, the narcissism scale showed a mean score of 3.15 (*SD* = 0.69), with no significant differences between men and women (*p* = 0.431, *d* = 0.08; see Table 3).

**Table 3:** Comparison of means for independent samples by sex.

Variables	Group	Mean	*SD*	*t*	*df*	*d*	*p*
Infidelity	Female	1.24	0.36	−4.41	270.94	−0.45	**<0.001**
	Male	1.48	0.65				
Rejection	Female	1.75	0.64	1.66	395.97	0.17	0.098
	Male	1.66	0.51				
Emotional warmth	Female	2.51	0.79	0.60	394.27	0.06	0.546
	Male	2.46	0.72				
Overprotection	Female	2.39	0.59	1.45	395.43	0.14	0.148
	Male	2.31	0.53				
Narcissism	Female	3.18	0.63	0.79	352.89	0.08	0.431
	Male	3.12	0.76				

Significant *p*-values are presented in bold.

### 3.2 Conditional mediation analysis

The findings from the mediation analysis reveal significant relationships among some of the studied variables, considering parenting styles as predictor variables, narcissistic traits as the mediator, infidelity behavior as the outcome variable, and sex assigned at birth as the grouping variable. Firstly, the full (average) model was statistically significant (*p* < 0.001, *R*^2^ = 0.12). A *post-hoc* power analysis was conducted using the observed model fit (*R*^2^ = 0.12, *n* = 399), yielding an estimated effect size of *f*^2^ = 0.14 and an achieved power of approximately 0.99. This indicates adequate sensitivity to detect medium-sized mediation effects.

The results of the mediation analysis on the full sample reveal that parental rejection has both a total effect (β = 0.17, *p* = 0.012) and a direct effect (β = 0.15, *p* = 0.024) on infidelity scores. Additionally, narcissism mediates the relationship between parental emotional warmth and engaging in infidelity during adulthood (β = 0.07, *p* = 0.002; see Table 4).

**Table 4:** Average mediation analysis.

Type	Effect	Estimate	SE	95% *CI*		β	*z*	*p*
				*LL*	*UL*			
Indirect	Rejection ⇒ Narcissism ⇒ Infidelity	0.02	0.01	−0.01	0.04	0.02	1.38	0.167
	Emotional warmth ⇒ Narcissism ⇒ Infidelity	0.05	0.02	0.02	0.08	0.07	3.12	**0.002**
	Overprotection ⇒ Narcissism ⇒ Infidelity	0.01	0.01	−0.02	0.02	0.00	0.04	0.966
Component	Rejection ⇒ Narcissism	0.12	0.08	−0.04	0.27	0.10	1.5	0.133
	Narcissism ⇒ Infidelity	0.13	0.04	0.06	0.21	0.18	3.53	**<0.001**
	Emotional warmth ⇒ Narcissism	0.36	0.05	0.25	0.46	0.39	6.62	**<0.001**
	Overprotection ⇒ Narcissism	0.01	0.07	−0.13	0.14	0.01	0.04	0.966
Direct	Rejection	0.13	0.06	0.02	0.25	0.15	2.26	**0.024**
	Emotional warmth	−0.06	0.04	−0.14	0.02	−0.09	−1.38	0.168
	Overprotection	−0.02	0.05	−0.13	0.08	−0.02	−0.42	0.673
Total	Rejection	0.15	0.06	0.03	0.27	0.17	2.5	**0.012**
	Emotional warmth	0.01	0.04	−0.09	0.08	−0.01	−0.12	0.904
	Overprotection	−0.02	0.05	−0.13	0.08	−0.02	−0.42	0.672

CI, Confidence Interval; LL, Lower Limit; UL, Upper Limit. Significant *p*-values are presented in bold.

**Figure 2:**
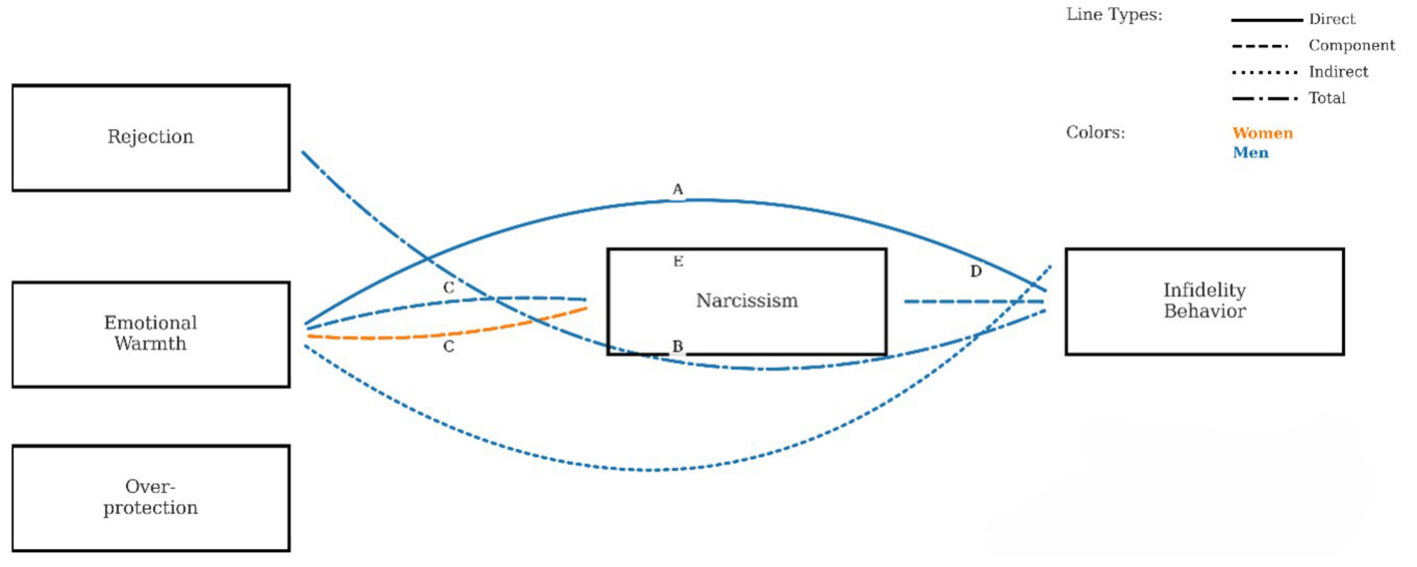
Significant paths reported for men and women. A, total effect (men, β = 0.21, *p* = 0.032); B, direct effect (men, β = −0.19, *p* = 0.039); C, component effect (women, β = 0.29, *p* < 0.001; men, β = 0.49, *p* < 0.001); D, component effect (men, β = 0.27, *p* < 0.001); E, indirect effect (men, β = 0.13, *p* < 0.001).

In the female group, emotional warmth had a positive and significant effect on narcissistic traits (β = 0.29, *p* < 0.001). However, no statistically significant direct or indirect effects were found in the other pathways for this group (*p* > 0.05; see Table 5) (Figure 2).

**Table 5:** Conditional mediation analysis for females.

Type	Effect	Estimate	SE	95% *CI*		β	*z*	*p*
				*LL*	*UL*			
Indirect	Rejection ⇒ Narcissism ⇒ Infidelity	0.01	0.01	−0.01	0.02	0.01	0.83	0.408
	Emotional warmth ⇒ Narcissism ⇒ Infidelity	0.02	0.01	0.01	0.04	0.02	1.50	0.134
	Overprotection ⇒ Narcissism ⇒ Infidelity	0.01	0.01	−0.01	0.01	0.01	0.18	0.857
Component	Rejection ⇒ Narcissism	0.10	0.11	−0.11	0.31	0.08	0.96	0.337
	Narcissism ⇒ Infidelity	0.06	0.04	−0.01	0.14	0.08	1.64	0.101
	Emotional warmth ⇒ Narcissism	0.26	0.07	0.12	0.4	0.29	3.72	**<0.001**
	Overprotection ⇒ Narcissism	0.02	0.09	−0.17	0.2	0.01	0.18	0.857
Direct	Rejection	0.10	0.08	−0.05	0.26	0.12	1.31	0.190
	Emotional warmth	0.01	0.05	−0.09	0.12	0.02	0.24	0.808
	Overprotection	−0.01	0.07	−0.15	0.13	−0.01	−0.15	0.881
Total	Rejection	0.11	0.08	−0.05	0.27	0.12	1.36	0.173
	Emotional warmth	0.03	0.05	−0.08	0.14	0.04	0.54	0.589
	Overprotection	−0.01	0.07	−0.15	0.13	−0.01	−0.13	0.895

CI, Confidence Interval; LL, Lower Limit; UL, Upper Limit. Significant *p*-values are presented in bold.

In contrast, for the male group, a parenting style characterized by rejection was associated with higher levels of infidelity-related behavior at the level of the total effect (β = 0.21, *p* = 0.032), although the corresponding direct effect did not reach significance (β = 0.18, *p* = 0.061). Emotional warmth showed the opposite pattern: a direct negative association with infidelity-related behavior (β = −0.19, *p* = 0.039), alongside an indirect positive association operating through narcissistic traits (β = 0.13, *p* < 0.001). Because these two paths ran in opposite directions, the total effect of emotional warmth was not significant (β = −0.06, *p* = 0.528), consistent with a pattern of inconsistent mediation rather than a straightforward protective effect. At the level of individual component paths, subclinical narcissistic traits significantly predicted higher infidelity-related behavior (β = 0.27, *p* < 0.001), and emotional warmth significantly predicted higher subclinical narcissistic traits (β = 0.49, *p* < 0.001; see Table 6).

**Table 6:** Conditional mediation analysis for males.

Type	Effect	Estimate	SE	95% *CI*		β	*z*	*p*
				*LL*	*UL*			
Indirect	Rejection ⇒ Narcissism ⇒ Infidelity	0.03	0.02	−0.02	0.07	0.03	1.13	0.257
	Emotional warmth ⇒ Narcissism ⇒ Infidelity	0.09	0.02	0.05	0.14	0.13	3.87	**<0.001**
	Overprotection ⇒ Narcissism ⇒ Infidelity	0.00	0.02	−0.04	0.04	0.00	−0.11	0.913
Component	Rejection ⇒ Narcissism	0.13	0.11	−0.09	0.36	0.11	1.16	0.247
	Narcissism ⇒ Infidelity	0.21	0.04	0.13	0.28	0.27	5.43	**<0.001**
	Emotional warmth ⇒ Narcissism	0.45	0.08	0.29	0.61	0.49	5.53	**<0.001**
	Overprotection ⇒ Narcissism	−0.01	0.1	−0.21	0.19	−0.01	−0.11	0.913
Direct	Rejection	0.16	0.09	−0.01	0.33	0.18	1.87	0.061
	Emotional warmth	−0.13	0.06	−0.26	−0.01	−0.19	−2.07	**0.039**
	Overprotection	−0.03	0.08	−0.18	0.12	−0.04	−0.44	0.663
Total	Rejection	0.19	0.09	0.02	0.36	0.21	2.14	**0.032**
	Emotional warmth	−0.04	0.06	−0.16	0.08	−0.06	−0.63	0.528
	Overprotection	−0.04	0.08	−0.19	0.12	−0.04	−0.46	0.649

CI, Confidence Interval; LL, Lower Limit; UL, Upper Limit. Significant *p*-values are presented in bold.

## 4 Discussion

This study investigated (1) the prevalence of infidelity behaviors, (2) the predictive role of perceived parenting styles during childhood on adult infidelity, (3) the mediating role of subclinical narcissistic traits, and (4) potential gender differences in these associations. The research was conducted among university students in Honduras, providing novel evidence from a population that has been largely overlooked and underrepresented in the academic literature (Polanco and Mayorga, 2025).

The findings revealed a consistent pattern of higher self-reported extradyadic behaviors, cognitions, and desires among men relative to women across all IMIN items examined (see Table 2). The observed prevalence of infidelity behaviors was somewhat smaller than that observed in prior research. Arantes et al. (2020) report that extramarital affairs occur in at least 25.55% of couples, with a higher perpetration committed by men. Similarly, men are more likely to have had sexual relations with someone other than their partner (19.4% compared to 13.9% for women) (Vail-Smith et al., 2010). These differences align with our results, indicating consistent gender differences in both behavioral and attitudinal indicators of infidelity.

This difference between men and women may relate to the fact that men tend to have lower expectations than women regarding relationships and are therefore less inclined to favor exclusivity (Boekhout et al., 2003). Studies suggest that perceiving high levels of power amplifies infidelity behaviors, as it generates a sense of control over one's partner, which in turn may lead men to believe that it is possible to replace their current partner with a more desirable one (Birnbaum et al., 2025). Moreover, studies suggest that men interpret specific cues as more inviting to sexual activity than women, which may contribute to their greater propensity for sexual infidelity (Lindgren et al., 2008). Women, by contrast, appear less concerned with breaking exclusivity within their primary relationship. This may be explained by the fact that they often have a broader social network than men, and may view extradyadic relationships in less sexual terms. However, it is important to note that affective commitment and power within a relationship do not necessarily imply causality. That is, depending on various factors, a person with lower commitment may have less power, just as holding a higher position of power may be associated with lower commitment to the relationship (Lindová et al., 2021).

Beyond general sex differences reported in the literature, the present pattern may also reflect the Honduran sociocultural setting. In contexts where traditional masculinity norms remain influential, men may encounter greater social tolerance for extradyadic desire and behavior, whereas women may face stronger sanctions for comparable conduct. Under these conditions, the higher male endorsement of infidelity-related behaviors observed in the present study may reflect not only dispositional or evolutionary factors, but also a gendered normative structure that differentially regulates sexual behavior in men and women. At the same time, more restrictive expectations surrounding women's sexuality may also contribute to lower disclosure of such behaviors among women, even in anonymous surveys.

Likewise, it was found that parental rejection during childhood predicted a higher engagement in infidelity in adulthood. The absence of warmth or the presence of excessive control in childhood may foster the development of anxious or avoidant attachment styles, which hinder the formation of secure bonds and, consequently, increase the risk of engaging in problematic relationship behaviors, such as infidelity (Ghiasi et al., 2024).

Furthermore, for the aggregated sample of men and women, subclinical narcissism mediated the relationship between emotional warmth and engaging in adult infidelity. Parenting characterized by overvaluation, permissiveness, or excessive praise may foster grandiose narcissistic traits, such as inflated self-importance and an unrealistically positive self-view (Van Schie et al., 2020; Green et al., 2020). This pattern was consistent with previous studies (Horton et al., 2006), which proposed that highly involved but overly indulgent parenting can foster an unhealthy narcissistic self. The findings suggest that emotionally warm and overvaluing parenting styles may promote narcissism by reinforcing an exaggerated sense of self-worth (Brummelman et al., 2015), which in turn increases the likelihood of infidelity.

Overall, the presence of subclinical narcissistic traits allows for the prediction of infidelity behavior, as individuals with high levels of subclinical narcissism tend to hold permissive and non-exclusive beliefs about relationships, where the absence of commitment and a favorable attitude toward infidelity do not represent a moral conflict for them. Additionally, narcissists' constant need for validation from others may lead them to engage with multiple partners as a means to reinforce their ego and sustain their self-image, detaching themselves from moral responsibility, which contributes to minimizing the perceived severity of their actions (Besser and Zeigler-Hill, 2024).

However, the distinct path models for men and women indicate different underlying mechanisms. For men, emotional warmth was positively related to narcissistic traits, which in turn increased the likelihood of infidelity. Yet, emotional warmth also showed a direct negative association with infidelity, suggesting that it may function as both a risk and protective factor. In contrast, the model for women did not significantly predict infidelity, possibly reflecting the more complex and multifaceted nature of female infidelity. Previous literature can provide insight into these findings, suggesting that women exhibit evolutionary tendencies to engage in infidelity to balance the pleasure derived from physical attraction to lovers while securing genetics, parental care, and fertility with their primary partner (Murphy et al., 2024).

The present findings may also be interpreted in light of the Honduran sociocultural context, a relatively underexamined setting in the literature on sexuality and intimate relationships. In Honduras, available evidence suggests that sexuality and couple dynamics remain shaped by persistent gender asymmetries, including norms that regulate girls' and women's roles and sexuality (Pacheco-Montoya et al., 2022), as well as by family socialization patterns in which hierarchy, control, and punitive discipline continue to play a relevant role. Local studies have also documented support for corporal punishment and parenting configurations characterized by high control and verbal-physical discipline (Landa-Blanco et al., 2026; Cárcamo-Zepeda et al., 2026), indicating that coercive and authority-centered forms of child-rearing remain part of the developmental environment for many Hondurans. Although not directly measured, these contextual factors provide a meaningful framework for interpreting the observed sex differences and parenting-related correlates of infidelity-related behavior. The findings may therefore reflect not only general psychological processes, but also broader sociocultural influences on adult intimacy in Honduras. These patterns are also consistent with honor culture frameworks, in which men's extradyadic behavior can function as a status-defending display and women's infidelity is judged as disproportionately damaging to male reputation, increasing the reputational cost of both engaging in and disclosing extradyadic involvement for women specifically (Dietrich and Schuett, 2013; Vandello and Cohen, 2008, 2003). These explanations should be understood as theoretically informed rather than empirically tested within the present study.

Despite the relevance of the findings, the current study has limitations that must be considered. First, the use of non-probabilistic convenience sampling, in which only those willing to participate were included, limited the representativeness of the results. Second, the possibility of self-report biases is also acknowledged, given the sensitive nature of infidelity, which may have led to underreporting due to social desirability. Third, the corresponding SD-4 subscale focuses on grandiose narcissism. Therefore, by limiting the analysis to a single dimension, other expressions of narcissism may have been overlooked, especially those related to insecurity or hypersensitivity. Fourth, although the IMIN behavior subscale captures a broad range of infidelity-related behaviors, desires, and emotional experiences, it does not directly assess whether each behavior violated an explicit relationship agreement or whether the partner considered the behavior infidelity. Therefore, the outcome should be interpreted as infidelity-related behaviors and desires rather than as a direct measure of confirmed partner betrayal in every case. Fifth, although statistically significant, the model accounted for only 12% of the explained variance in infidelity scores. Sixth, the sample was drawn exclusively from Honduras, and thus, caution is warranted when generalizing the results to other national or cultural settings. Seventh, the findings should be interpreted as relational rather than causal. Although the indirect associations are consistent with the proposed theoretical model, longitudinal research is needed to determine whether recalled parenting styles precede narcissistic traits and whether these traits subsequently contribute to infidelity-related behaviors and desires.

To address these limitations, future research should employ larger probabilistic samples, locally validate the instruments, and consider longitudinal designs to analyze the stability of these relationships over time. Likewise, it would be valuable to explore other potential mediators or moderators, such as emotional regulation, partner satisfaction, or attitudes toward monogamy, which could enrich the understanding of infidelity and guide more precise clinical interventions.

In summary, men consistently reported higher levels of behavioral and attitudinal infidelity than women, including greater engagement in and desire for extradyadic relationships. These patterns suggest distinct gender differences in relational dynamics and the expression of infidelity-related behaviors, as evidenced in the conditional mediation analysis. In this sense, infidelity behavior was not predicted in women with the variables considered in this study. However, in men, parental rejection increased the tendency toward infidelity, while parental emotional warmth decreased it. Indirectly, this relationship was mediated by narcissistic traits, which increased such a tendency. These results point to distinct gender patterns linking received parenting styles, narcissism, and infidelity. In this line, clinical practice could be enriched by sex-specific models for addressing infidelity. For instance, approaches such as Cognitive-Existential Therapy, Cognitive-Behavioral Therapy, Cognitive Processing Therapy, and Acceptance and Commitment (Gorijan-Mehlabani et al., 2023; Hines, 2025).

## Data Availability

The datasets presented in this study can be found in online repositories. The names of the repository/repositories and accession number(s) can be found below: the dataset that supports the findings of this study is available in the Zenodo repository at: 10.5281/zenodo.17460580.
